# Patterns of C-reactive protein trends during clozapine titration and the onset of clozapine-induced inflammation: a case series of weekly and daily C-reactive protein monitoring

**DOI:** 10.3389/fpsyt.2024.1366621

**Published:** 2024-02-21

**Authors:** Yuki Kikuchi, Hiroaki Tanifuji, Sota Ueno, Yoshifumi Onuma, Masatomo Goto, Masato Ishihara, Takeshi Toraiwa, Hiroshi Komatsu, Hiroaki Tomita

**Affiliations:** ^1^ Department of Psychiatry, Graduate School of Medicine, Tohoku University, Sendai, Miyagi, Japan; ^2^ Department of Psychiatry, Kodama Hospital, Ishinomaki, Miyagi, Japan; ^3^ Department of Pharmacy, Kodama Hospital, Ishinomaki, Miyagi, Japan; ^4^ Department of Psychiatry, Tohoku University Hospital, Sendai, Miyagi, Japan

**Keywords:** C-reactive protein, DRESS, fever, myocarditis, pneumonia

## Abstract

**Background:**

International guidelines for clozapine titration recommend measuring C-reactive protein (CRP) weekly for 4 weeks after clozapine initiation to prevent fatal inflammatory adverse events, including myocarditis. However, limited evidence exists regarding whether weekly CRP monitoring can prevent clozapine-induced inflammation.

**Aims:**

We examined the relationship between CRP trends and the development of clozapine-induced inflammation. We also explored the usefulness and limitations of CRP monitoring during clozapine titration.

**Method:**

This study presents 17 and 4 cases of weekly and daily CRP monitoring during clozapine initiation, respectively.

**Results:**

Among 17 patients with weekly CRP measurements, 7 had fever. Elevated CRP levels were detected before the onset of fever in two of the seven patients. Of the five remaining patients, the CRP levels on a previous test had been low; however, the fever developed suddenly. Of the 10 patients with no fever under weekly CRP monitoring, three had elevated CRP levels >3.0 mg/dL. Refraining from increasing the clozapine dose may have prevented fever in these patients. Among four patients with daily CRP measurements, two became febrile. In both cases, CRP levels increased almost simultaneously with the onset of fever.

**Conclusion:**

Weekly and daily CRP monitoring during clozapine titration is valuable for preventing clozapine-induced inflammation, assessing its severity, and guiding clozapine dose adjustments. Weekly CRP monitoring may not adequately predict clozapine-induced inflammation in some cases. Consequently, clinicians should be aware of the sudden onset of clozapine-induced inflammation, even if CRP levels are low. Daily CRP monitoring is better for detecting clozapine-induced inflammation.

## 1 Introduction

Clozapine is the most effective antipsychotic for treatment-resistant schizophrenia; however, it is underused and overlooked owing to its various adverse effects (1, 2). Clinicians should titrate clozapine with caution because myocarditis, pneumonia, and other adverse inflammatory effects commonly occur within 4 weeks of starting clozapine treatment and are frequently fatal (3). Recently, de Leon et al. published an international guideline for clozapine titration that considers factors that influence clozapine metabolism, such as ethnicity, sex, and smoking, to prevent the adverse inflammatory effects associated with clozapine (4). The guideline recommends a gradual increase in clozapine dosage for Asians, who are lower clozapine metabolizers than Caucasians. In fact, our previous study suggested that a slower clozapine titration rate, based on the guideline-recommended titration speed, reduces the adverse inflammatory effects of clozapine in Japanese patients (5, 6). According to the guideline, C-reactive protein (CRP) should be measured weekly for 4 weeks after commencing clozapine, and the clozapine dosage should remain unchanged if the CRP level rises.

The incidence of inflammatory complications associated with clozapine varies across countries, with a high incidence in Australia (7). In Australia, standard practice involves measuring CRP and troponin levels at the start of clozapine administration (8–11), and thereafter in weeks 1,2,3,4,6 and 8, and every 6 months. The Australian protocol recommends switching to daily CRP monitoring if the CRP level exceeds 5 mg/dL and discontinuing clozapine if the CRP level exceeds 10 mg/dL (10). CRP levels of 5 and 10 mg/dL were determined based on data from patients who had myocarditis (10). When CRP reaches these levels, patients frequently experience symptoms of clozapine-induced inflammation (8, 12, 13). CRP monitoring before fever onset is useful, as it has been reported that approximately 20% of patients with clozapine-induced myocarditis are afebrile (10).

In our previous study, we found that clozapine-induced fever was frequent in Japanese patients. Approximately 30% of patients experienced this side effect under the dosing protocol stated in clozapine’s Japanese package insert. Even with a slower titration than what is recommended in the guideline, approximately 10% still had fever (5). In our more severely ill patients with myocarditis, pneumonia, or drug reaction with eosinophilia and systemic symptoms syndrome, a fever of 39°C or higher and a CRP level of 10 mg/dL or higher are common (5). However, some patients experience myocarditis or pneumonia before their CRP level reaches 5 mg/dL (5, 6, 14). The de Leon et al. guideline was intended to detect elevated CRP levels and halt clozapine titration to prevent the onset and severity of clozapine-induced inflammation (4).

However, no clear criteria exist for clinicians to determine when to cease increasing the clozapine dosage to avoid clozapine-induced inflammation (15). Furthermore, the extent to which weekly CRP monitoring can be used to detect elevated CRP levels before the onset of clozapine-induced inflammation remains unclear. Moreover, limited evidence exists regarding whether lowering the clozapine dose can reduce adverse inflammatory effects when elevated CRP levels are detected. Therefore, to answer these questions, prospective studies are needed to determine the exact CRP level that warrants clinical attention and to examine whether the frequency of inflammatory adverse effects of clozapine changes with different dose adjustments when the CRP level is detectable.

In this study, we aimed to investigate in a practice-based setting, whether CRP is a useful marker of inflammation (added to or instead of body temperature) and whether CRP could have a supportive role in preventing inflammation. For this purpose, we present CRP trends in 17 patients with weekly CRP measurements according to the guidelines and four patients with daily CRP measurements from the start of clozapine administration. Through these cases, we examined the relationship between CRP trends and the development of clozapine-induced inflammation. We also explored the usefulness and limitations of CRP monitoring during clozapine titration.

## 2 Method

All patients in this case series were Japanese individuals with treatment-resistant schizophrenia who were first initiated on clozapine. In Japan, the initiation of clozapine is mandated to be performed in an inpatient setting. As a routine practice, body temperature was measured in the armpit at least twice a day (at 6 am and 2 pm); for patients who had fever, it was measured at least four times a day (additionally at 9 am and 7 pm). The highest body temperature of the day is listed in the Tables. Weekly CRP measurements were performed simultaneously with blood tests to determine the neutrophil count required for clozapine administration. The CRP levels were measured using the latex immunoturbidimetric method (measurable range: 0.01–42 mg/dL; CRP-N, Shino-Test Corporation, Tokyo, Japan). Daily CRP measurements were taken at approximately 10 am using a Yumizen M100 Banalyst (measurable range 0.1–20 mg/dL; Kyoto, Japan) via finger capillary blood sampling. Previous studies have not provided clear parameters for the threshold of elevated CRP that poses a risk for the development of clozapine-induced inflammation (15). However, previous studies have indicated that the average CRP level is approximately 0.5 mg/dL in patients using clozapine (16–19). Based on our experience, we set a CRP value of 1.0 mg/dL or higher as the alert level, considering that some patients have a fever when their CRP reaches 1.0 mg/dL or higher (5). In the four patients whose CRP levels were measured daily, clozapine blood levels were measured at the trough time point on day 8 (all four patients were administered clozapine once a day in the evening, and blood was collected at approximately 3 pm the next day). The measurement of clozapine blood levels was outsourced to LSI Medience Corporation (Tokyo, Japan) and was performed using liquid chromatography-mass spectrometry.

Written informed consent for daily CRP measurements was obtained from all four target patients. The authors assert that all procedures contributing to this work comply with the ethical standards of the relevant national and institutional committees on human experimentation and with the Helsinki Declaration of 1975, as revised in 2008. All procedures involving patients were approved by the Tohoku University Hospital Ethics Review Board (approval ID: 2023-1-509). Written informed consent was obtained from all patients. This study does not follow the CARE guidelines because it aims to show the relationship between CRP trends and fever onset in 21 patients, which differs from the CARE guidelines’ narrative description of a single patient or a small number of patients.

## 3 Results

The clinical characteristics and psychopathological descriptions of the patients are summarized in **Supplementary Table 1**. All patients were non-smokers during clozapine titration and were not taking any cytochrome P450 1A2 inhibitors such as fluvoxamine, ciprofloxacin, or oral contraceptives. In addition, none of the patients had any physical symptoms or vital signs suggestive of inflammation during clozapine initiation. Similarly, none of the patients had any history of physical disease that could cause inflammation.

### 3.1 Weekly CRP trends in 10 cases without fever on clozapine titration

**Table 1** shows the weekly CRP trends for the 10 patients who had no fevers upon clozapine titration. The patients were asymptomatic during the entire treatment course. Three patients (Cases 1–3) maintained CRP levels below 1.0 mg/dL during the entire period. Additionally, four patients (Cases 4–7) exhibited elevated CRP levels >1.0 mg/dL; however, these did not persist for more than 1 week. In three patients (Cases 8–10), CRP levels >1.0 mg/dL remained consistently high for the entire 2-week duration of CRP measurement.

**Table 1:** Weekly CRP trends in 10 cases without fever on clozapine titration.

Case	Age	Sex	BMI	Concomitant drugs	Day	1	8	15	19	22	23	24	25	29	30	31	32	36	37	38	39	43
1	48	F	23.8	Olanzapine 20 mg, Paliperidone 12 mg, Li 600 mg, Flunitrazepam 1 mg	CRP	0.06	0.06	0.06		0.06				0.09				0.22				0.09
					BT	36.4	36.7	36.8	36.5	36.5	36.4	36.6	36.5	36.5	36.3	36.4	36.6	36.4	36.5	36.5	36.9	36.7
					Dose	12.5	50	75	75	100	100	100	100	125	125	125	125	150	150	150	150	150
2	62	F	21.8	Haloperidol decanoate 100 mg monthly, CP 50 mg	CRP	0.03	0.03	0.07		0.03				0.11							0.06	
					BT	36.7	36.2	36.7	36.6	36.4	36.9	36.7	36.8	36.9	36.5	36.6	36.5	36.6	36.6	36.5	36.8	36.4
					Dose	12.5	50	75	75	75	75	75	75	100	100	100	100	150	150	150	150	175
3	43	M	21.0	Haloperidol 12 mg, LP 150 mg, Flunitrazepam 4 mg	CRP	0.05	0.28	0.09		0.12				0.05				0.17				0.94
					BT	36.6	36.5	36.4	36.5	36.0	36.4	36.4	36.4	36.4	36.3	36.3	36.3	36.0	36.4	36.2	36.5	36.3
					Dose	12.5	50	75	75	100	100	100	100	100	125	125	125	125	150	150	150	150
4	36	M	30.0	Quetiapine 500 mg, LP 100 mg, Flunitrazepam 2 mg, Triazolam 0.25 mg	CRP	0.18	0.13	0.14		**1.26**				0.52				0.14				0.29
					BT	36.5	36.4	36.7	36.2	36.3	36.5	36.4	36.1	36.4	36.6	36.2	36.3	36.1	35.9	36.0	36.2	36.4
					Dose	12.5	25	75	75	100	100	100	100	125	125	125	125	150	150	150	150	150
5	22	M	25.6	Olanzapine 20 mg, LP 200 mg, Flunitrazepam 2 mg	CRP	0.13	0.18	0.15				0.11				**1.6**				0.3		
					BT	36.9	36.2	36.3	36.3	36.1	36.0	35.9	36.3	36.4	36.4	36.6	36.7	36.1	36.1	36.0	36.9	36.2
					Dose	12.5	50	75	75	75	75	100	100	125	125	125	125	150	150	150	150	175
6	50	M	23.6	Olanzapine 20 mg, Quetiapine 150 mg, Lurasidone 40 mg, Li 1000 mg, Flunitrazepam 2 mg	CRP	0.04	0.16	0.13		**1.08**				0.08				0.81				0.05
					BT	36.3	36.6	36.6	36.6	36.3	36.2	36.4	36.2	36.6	36.4	36.6	36.6	36.6	36.6	36.4	36.9	36.8
					Dose	12.5	37.5	75	75	75	87.5	87.5	87.5	87.5	100	100	100	100	100	100	125	125
7	42	M	25.9	Paliperidone 12 mg, Blonanserin 24 mg, Flunitrazepam 2 mg	CRP	0.14	**1.49**	0.65		**1.63**				0.46				0.52				0.74
					BT	36.3	36.2	36.4	36.2	36.3	36.5	36.2	36.0	36.1	36.5	36.4	36.0	36.4	36.2	36.8	36.3	36.7
					Dose	12.5	75	150	175	200	200	200	200	200	200	200	200	300	300	300	300	300
8	52	M	19.9	Risperidone 5 mg, Ramelteon 8 mg	CRP	0.09	0.12	**1.0**	**4.04**		**1.23**				0.67				0.1			
					BT	36.3	36.4	36.3	36.6	36.4	36.1	36.2	36.5	36.7	36.2	36.2	36.4	36.7	36.2	36.6	36.4	36.3
					Dose	12.5	50	75	75	75	75	75	75	75	100	100	100	100	100	100	100	100
9	33	F	19.2	Olanzapine 20 mg, Flunitrazepam 2 mg, Nitrazepam 10 mg	CRP	0.04	0.03	0.8					0.08				**1.08**				**3.82**	
					BT	36.7	36.4	36.4	36.9	36.0	36.3	36.4	36.3	36.5	36.9	36.7	36.9	36.8	36.6	36.1	36.6	36.4
					Dose	12.5	50	75	75	75	75	100	100	100	125	125	125	150	150	150	150	150
10	24	M	21.1	Aripiprazole 30 mg, Nitrazepam 10 mg	CRP	0.03	0.1	**7.63**		**2.9**				**1.06**				0.86				**1.34**
					BT	36.5	36.6	36.6	36.1	36.5	36.7	36.7	36.7	36.4	36.0	36.6	36.9	36.6	36.6	36.6	36.5	36.6
					Dose	12.5	50	50	50	75	75	75	100	125	125	125	125	175	175	175	175	200

BMI, body mass index; BT, body temperature; CP, chlorpromazine; CRP, C-reactive protein; Li, lithium carbonate; LP, levomepromazine0.

Units are as follows: CRP (mg/dL), Dose (mg). CRP levels of 1.0 mg/dL or higher are shown in bold. Concomitant drugs indicate concomitant medication on day 1 of clozapine initiation.

In Case 8, the CRP rose to 1.0 mg/dL on day 15 and further rose to 4.04 mg/dL on day 19. Consequently, the clozapine dose was maintained at 75 mg/day for 2 weeks. Thus, the CRP levels spontaneously decreased and returned to their normal range. Eosinophilia of approximately 1000/μL was observed for approximately 3 weeks, starting on day 37.

In Case 9, the CRP levels increased to 1.08 mg/dL and 3.82 mg/dL on days 32 and 39, respectively. The clozapine dose was maintained at 150 mg/day thereafter, and the CRP levels spontaneously returned to normal (data not shown).

In Case 10, the CRP level suddenly increased to 7.63 mg/dL on day 15 without symptoms or vital sign changes. Clozapine was maintained at 50 mg/day, and on day 22, the dose was increased to 75 mg/day after the CRP level had dropped to 2.9 mg/dL. Eosinophilia of approximately 1000/μL was observed for approximately 2 weeks, starting on day 22. As the CRP level continued to decline, the clozapine dose was slowly increased, with no adverse inflammatory effects.

### 3.2 Weekly CRP trends in seven cases with fever on clozapine titration

**Table 2** shows the weekly CRP trends for the seven patients with fever during clozapine titration.

**Table 2:** Weekly CRP trends during clozapine titration in seven cases with fever.

Case	Age	Sex	BMI	Concomitant drugs	Day	1	8	14	15	16	17	18	19	20	21	22	23	25	26	28	29	30	36	43
11	69	F	25.1	Olanzapine 20 mg, Risperidone 0.5 mg, Flunitrazepam 1 mg	CRP	0.28	0.43	**1.93**			**5.75**					**2.43**					0.24		0.2	
					BT	37.2	36.6	**38.0**	**37.7**	**38.1**	36.9	36.8	36.7	37.1	37.0	37.0	36.6	36.8	37.0	37.3	36.8	36.4	36.5	36.7
					Dose	12.5	75	100	100	100	100	100	100	100	100	100	100	100	100	100	100	100	100	100
12	60	M	26.2	Olanzapine 20 mg, Risperidone 8 mg, LP 125 mg, Flunitrazepam 2 mg, Nitrazepam 10 mg	CRP	0.25	0.3	**1.27**			**1.92**					**4.81**					0.89		0.52	0.99
					BT	36.5	36.2	36.1	36.9	36.8	**38.8**	**39.0**	**39.3**	36.7	36.8	36.2	36.8	36.6	36.9	36.4	36.9	36.3	36.6	36.6
					Dose	12.5	50	100	100	100	50	50	50	50	75	75	75	100	100	125	125	125	175	250
13	25	M	20.0	Olanzapine 20 mg, Blonanserin patch 80 mg, Nitrazepam 10 mg	CRP	0.4	0.62		**1.84**							**2.14**			**8.72**		0.92		**1.94**	**2.22**
					BT	36.6	36.5	36.6	36.7	36.8	36.7	36.8	36.7	36.8	36.7	36.5	36.5	**37.8**	36.8	37.2	36.9	36.7	36.8	36.4
					Dose	12.5	50	50	50	75	75	75	75	75	75	75	100	0	25	25	50	50	50	50
14	43	F	19.7	Blonanserin 24 mg, Flunitrazepam 2 mg, Brotizolam 0.5 mg	CRP	0.03	0.43	0.06		**4.02**		**2.37**				0.17					0.12		0.69	
					BT	36.6	36.4	36.3	**37.9**	**38.2**	37.2	36.3	36.6	36.3	36.2	36.0	36.6	36.8	36.8	36.3	36.4	36.6	36.8	36.7
					Dose	12.5	50	75	75	0	0	0	0	0	0	12.5	12.5	12.5	12.5	12.5	25	25	12.5	12.5
15	35	F	26.7	Risperidone 6 mg, LP 50 mg, Flunitrazepam 2 mg, Triazolam 0.25 mg	CRP	0.08	0.05		0.28							**3.16**		**1.19**			0.15		0.08	0.06
					BT	36.4	36.9	36.4	**37.6**	37.2	**37.9**	36.8	36.8	**37.9**	**38.2**	37.3	37.1	36.6	36.2	36.9	36.6	36.3	36.8	36.8
					Dose	12.5	50	50	75	75	75	75	75	75	75	75	75	75	75	75	75	100	100	100
16	37	F	15.9	Olanzapine 5 mg, Quetiapine 300 mg, Li 400 mg, Nitrazepam 5 mg	CRP	0.09	0.66		0.17						**6.42**					0.66				
					BT	36.8	36.6	36.5	36.8	36.6	36.7	36.8	37.3	**39.2**	**37.6**	**37.9**	37.2	36.7	36.8	37.0	37.1	36.8	36.9	36.7
					Dose	12.5	25	25	50	50	50	50	50	50	12.5	0	0	0	0	0	0	0	0	0
17	30	M	21.2	Flunitrazepam 2 mg, Brotizolam 0.5 mg	CRP	0.05	0.04		0.06							**8.21**					0.25		0.8	0.2
					BT	37.4	36.5	36.2	36.1	36.3	36.0	36.5	36.3	36.5	36.8	**38.8**	36.2	36.6	36.7	36.6	36.5	36.2	36.6	36.5
					Dose	12.5	12.5	12.5	12.5	25	25	25	25	25	25	0	6.25	6.25	6.25	6.25	6.25	6.25	12.5	12.5

Units are as follows: CRP (mg/dL), BT(°C), and Dose (mg). CRP levels of 1.0 mg/dL or higher and BT of 37.5°C or higher are shown in bold. Concomitant drugs indicate concomitant medication on day 1 of clozapine initiation.

BMI, body mass index; BT, body temperature; CRP, C-reactive protein; Li, lithium carbonate; LP, levomepromazine.

The CRP level in Case 11 was 0.43 mg/dL on day 8; however, on day 14, the patient suddenly developed a fever of 38°C; the body temperature from day 9 to day 13 was < 37°C (data not shown), and the CRP level increased to 1.93 mg/dL. Clozapine was maintained at 100 mg/day, and the fever subsided over 3 days. As the CRP level remained elevated at 5.75 mg/day on day 17, clozapine was not increased. The CRP level subsequently dropped to 0.24 mg/dL on day 29. Mildly elevated eosinophil counts were observed on day 35 (849/μL).

In Case 12, the CRP level increased to 1.27 mg/dL on day 14. On day 17, the CRP level rose to 1.92 mg/dL, and the patient developed a fever. Clozapine was increased three days after the subsidence of the fever. The CRP level rose to 4.81 mg/dL on day 22; however, the patient did not develop a fever again, and the CRP level decreased to 0.89 mg/dL on day 29.

In Case 13, the CRP level increased to 1.84 mg/dL and 2.14 mg/dL on days 15 and 22, respectively. On day 25, the patient became febrile with a temperature of 37.8°C. Clozapine was discontinued for that day only. The next day, the CRP level was high at 8.72 mg/dL; however, the fever resolved. Subsequently, the clozapine dose was gradually increased.

In Case 14, the CRP level was low on day 14 (0.06 mg/dL); however, the patient suddenly became febrile the next day. On day 16, the fever continued to persist, and the CRP level further increased to 4.02 mg/dL. Clozapine was discontinued because the patient complained of distress owing to flu-like symptoms. After confirming a decrease in the CRP level, the clozapine dosage was resumed and gradually increased.

Case 15 had a CRP level of 0.28 mg/dL on day 15; however, this was accompanied by a low-grade fever. Since the patient was in good general condition, clozapine was continued at 75 mg/day. However, the temperature reached 38.2°C on day 21, and the CRP level increased to 3.16 mg/dL on day 22. Clozapine was maintained at 75 mg/day, and CRP levels gradually normalized. Subsequently, the clozapine dose was again increased.

Case 16 had a CRP level of 0.17 mg/dL on day 15 but suddenly developed a fever of 39.2°C on day 20. The following day, the CRP level rose to 6.42 mg/dL, and chest computed tomography revealed pneumonia with ground-glass opacities. Clozapine was discontinued, and the CRP levels normalized.

Case 17 had a CRP level of 0.06 mg/dL on day 15. However, on day 22, he suddenly developed a fever of 38.8°C along with an elevated CRP level of 8.21 mg/dL. Clozapine was discontinued for 1 day and restarted with a gradual dose increase.

### 3.3 Daily CRP trends during clozapine titration in four cases

**Table 3** shows four cases where the CRP level was measured daily while on clozapine titration. The CRP trends after day 30 are shown in **Supplementary Table 2**.

**Table 3:** Daily CRP trends during clozapine titration in four cases.

Case	Age	Sex	BMI	Concomitant drugs	Clozapine blood level on day 8	Day	1	2	3	4	5	6	7	8	9	10	11	12	13	14	15	16	17	18	19	20	21	22	23	24	25	26	27	28	29
18	49	M	27.5	Perospirone 48 mg, Lurasidone 80 mg, Flunitrazepam 1 mg, Nitrazepam 5 mg	56.2 ng/mL C/D ratio 1.12	CRP	0.1	0.1	0.1	0.1	0.1	0.1	0.1	0.1	**1.0**	0.6	0.3	**1.8 (am)** **4.4 (pm)**	**12.6 (am)** **>20 (pm)**	**19.7**	**7.0**	**5.2**	**3.5**	**2.1**	**1.1**	0.6	0.4	0.3	0.3	0.4	0.4	0.4	0.3	0.4	0.3
						BT	36.6	36.6	36.5	36.5	36.7	36.6	36.2	36.6	36.4	36.9	36.6	**38.5**	**38.8**	37.3	36.9	36.9	36.8	36.5	36.6	36.5	36.6	36.6	36.6	36.4	36.6	36.8	36.4	36.6	36.6
						Dose	12.5	25	25	25	50	50	50	75	75	100	100	50	0	0	0	0	0	0	0	0	12.5	25	25	25	25	25	25	50	50
19	17	F	22.9	Olanzapine 20 mg, Quetiapine 400 mg, Brotizolam 0.5 mg, Triazolam 0.5 mg	47.6 ng/mL C/D ratio 0.95	CRP	0.1	0.1	0.1	0.1	0.1	0.2	0.1	0.2	0.5	0.3	0.3	0.4	**3.1 (am)** **3.6 (pm)**	**3.0**	**5.2**	**5.0**	**3.5**	**2.2**	**1.2**	0.7	0.4	0.2	0.3	0.3	0.4	0.5	0.5	0.5	0.5
						BT	37.0	36.8	36.5	36.6	36.9	36.8	36.7	36.8	36.9	36.7	36.9	36.0	37.3	**38.2**	**39.6**	**38.7**	37.0	37.0	36.7	37.2	36.8	36.9	36.5	36.4	36.9	36.8	37.2	37.0	37.0
						Dose	12.5	25	25	25	50	50	50	75	75	100	100	125	125	50	0	0	0	0	12.5	25	25	25	50	50	50	50	75	75	75
20	42	F	36.1	Brexpiprazole 2 mg, Lurasidone 20 mg, Valproate 800 mg, Quetiapine 12.5 mg, Lemborexant 10 mg	28.7 ng/mL C/D ratio 2.30	CRP	0.5	0.5	0.6	0.5	0.5	0.6	0.5	0.6	0.7	0.8	0.6	0.6	0.6	0.7	0.8	0.8	0.8	0.7	0.7	0.7	0.9	0.8	0.8	**1.0**			0.8	0.6	0.5
						BT	35.4	36.4	36.2	35.9	36.2	36.3	36.5	35.9	36.2	36.4	35.6	36.3	35.6	36.3	36.1	36.2	36.3	36.0	36.1	36.4	35.6	36.3	36.3	36.5	36.4	36.1	36.0		36.1
						Dose	12.5	12.5	12.5	12.5	12.5	12.5	12.5	25	25	25	25	25	25	25	25	37.5	37.5	37.5	37.5	37.5	37.5	50	50	50	50	50	50	50	50
21	46	M	20.9	Asenapine 10 mg, Flunitrazepam 2 mg	53.0 ng/mL C/D ratio 1.06	CRP	0.1	0.1	0.1	0.1	0.1	0.1	0.1	0.1	0.1	0.1	0.1	0.1	0.1	0.1	0.1	0.1	0.2	0.2	0.1	0.2	0.3	0.2	0.1	0.1	0.1				
						BT	36.3	36.5	36.6	36.4	36.4	36.4	36.4	36.6	36.4	36.4	36.6	36.3	36.5	36.3	36.1	36.2	36.6	36.5	36.6	36.5	36.4	36.4	36.5	36.6	36.1				
						Dose	12.5	25	25	25	50	50	50	75	75	100	100	125	125	125	150	150	150	175	175	175	200	200	200	200	200				

Units are as follows: CRP (mg/dL), BT(°C), and Dose (mg). CRP levels of 1.0 mg/dL or higher and BT of 37.5°C or higher are shown in bold. Concomitant drugs indicate concomitant medication on day 1 of clozapine initiation.

BMI, body mass index; BT, body temperature; C/D ratio, concentration to dose ratio; CRP, C-reactive protein.

Case 18 had a low CRP level of 0.1 mg/dL until day 8, and it increased to 1.0 mg/dL on day 9. It initially declined but eventually increased to 1.8 mg/dL on the morning of day 12. In the afternoon of day 12, the patient became febrile, and the CRP level rose to 4.4 mg/dL. Clozapine was administered at 50 mg/day. On the morning of day 13, the CRP level remained elevated at 12.6 mg/dL, with persistent fever. In the afternoon, the CRP level was >20 mg/dL (immeasurable). Treatment with clozapine was discontinued. The day after discontinuation, the patient’s body temperature dropped to 37°C, and the CRP level gradually decreased. After confirming that CRP levels had returned to normal on day 21, clozapine was slowly resumed. Eosinophilia was observed on day 29 (day 29, 3570/μL; day 36, 1608/μL; day 43, 1239/μL; day 50, 601/μL; day 57, 1239/μL).

Case 19 had low CRP levels between 0.1 and 0.2 mg/dL until day 8 but increased slightly on day 9 to 0.5 mg/dL. On the morning of day 13, the CRP level was 3.1 mg/dL, and in the afternoon, it was 3.6 mg/dL. A low-grade fever of 37°C was observed at night. On the morning of day 14, the patient became febrile with a CRP level of 3.0 mg/dL. The patient complained of general malaise and headache; therefore, the clozapine dose was reduced to 50 mg/day. The next day, the temperature rose to 39.6°C, and the CRP level increased to 5.2 mg/dL. The clozapine blood level in the morning was 278 ng/mL with a concentration-to-dose ratio of 5.56; the result was known 1 week later because the testing was outsourced. Consequently, the clozapine treatment was discontinued. The day after discontinuation, the fever persisted, and the CRP level remained high. On day 17, the fever resolved to 37°C, and CRP showed a decreasing trend. Clozapine was resumed slowly on day 19. Eosinophilia was observed on day 36 (day 36, 737/μL; day 41, 1050/μL).

In Case 20, the risk of clozapine-induced inflammation was heightened by the concomitant use of valproic acid and obesity, resulting in a gradual increase in the clozapine dose. The baseline CRP level was slightly elevated at 0.5 mg/dL and reached 1.0 mg/dL on day 24; however, no fever occurred.

In Case 21, the CRP level persisted at 0.1 mg/dL and increased only slightly to 0.3 mg/dL on day 21. No fever occurred.

## 4 Discussion

### 4.1 Can weekly or daily CRP measurements detect elevated CRP before the onset of clozapine-induced inflammation?

In a study conducted in Australia, fever was not encountered in approximately 20% of the cases of myocarditis (10). Nevertheless, in both this case series and in our previous study encompassing 241 cases (5), severe inflammatory adverse effects of clozapine without fever were not observed; however, a few cases of liver injury, skin rash, and eosinophilia occurred without concomitant fever. Thus, fever is undoubtedly an important sign of clozapine-induced inflammation, and its prevention is important.

Of the seven patients with fever under weekly CRP monitoring, two (Cases 12 and 13) had detectably elevated CRP levels before fever onset. For the remaining five patients, no obvious signs of elevated CRP levels were detected before fever onset; however, the patients suddenly developed fever, and CRP level elevation was detected concurrently.

**Table 4** presents the cases of clozapine-induced myocarditis that were monitored with weekly CRP testing, as reported in previous studies (8, 12, 13). Of the 13 cases in the literature, elevated CRP levels were detected through weekly CRP monitoring before the onset of myocarditis in Case 2, as reported by Sandarsh et al.; Carswell et al. reported the same for Cases 2 and 4 in their investigation. In the remaining cases in the literature, no increase in CRP levels was observed prior to the onset of the disease; however, myocarditis developed abruptly, and the elevated CRP levels were detected concurrently.

**Table 4:** CRP trends in patients with clozapine-induced myocarditis whose CRP levels were monitored weekly in previous studies.

Author	Case	Symptom onset date	Symptoms at onset	Day 0	Day 7	Day 13	Day 14	Day 15	Day 16	Day 17	Day 18	Day 19	Day 20	Day 21	Day 22	Day 23	Day 28
Sandarsh et al. (2021) (12)	1	Day 13	Fever, diarrhea, flu-like symptoms	0.51	0.3		0.18	**6.06**									
	2	Day 10	Fever, diarrhea, tachycardia	0.71	**2.58**				**1.97**								
	3	Day 20	Fever, tachycardia	<0.1	0.2		0.10							**8.9**	**5.87**		
	4	Day 21	Complaint of “weakness”	0.05	0.05		0.08							0.12	0.19		0.16
McNutt et al. (2021) (13)	1	Day 12	Fever, symptoms of an upper respiratory infection, tachycardia		low		low							low			**10–15**
	2	After Day 14	Fever, tachycardia		low		low							**5–10**			
	3	Not described	tachycardia		low		low							**25–30**			
	4	Not described	Fever, tachycardia		low		low										**5–10**
Carswell et al. (2023) (8)	1	Day 14	Fever	0.13	0.17		**1.45**			**6.92**	**9.93**						
	2	Day 19	Fever	**2.43**	**1.85**		**1.0**					**17.33**	**22.58**	**19.92**	**18.70**		
	3	Day 18	Chest pain	0.04	0.05		0.15					**3.43**					
	4	No symptoms	No symptoms	**1.17**	0.56	**5.24**	**5.28**		**9.45 (am)** **12.40 (pm)**								
	5	Day 18	Fever, cough	0.6	0.76		0.79					**12.62**	**13.88**		**9.24**	**5.2**	

Units are as follows: CRP (mg/dL). CRP levels of 1.0 mg/dL or higher are shown in bold.

CRP, C-reactive protein.

In our current study, fever and elevated CRP levels were detected concomitantly in Cases 18 and 19, even with daily CRP measurements. Neither of these two cases had an elevated CRP level 1 week before the fever. These cases illustrate that weekly CRP monitoring does not predict the development of adverse inflammatory effects from clozapine.

The fact that many patients did not have fever and elevated CRP levels or that fever onset and elevated CRP detection were simultaneous may raise concerns about the need for weekly CRP monitoring. However, these findings do not negate the effectiveness of weekly CRP monitoring in preventing clozapine-induced inflammation. Confirming baseline CRP levels before clozapine administration is important because inflammation increases clozapine blood levels. In our case series, none of the patients had elevated baseline CRP levels before clozapine administration. In Cases 12 and 13, where elevated CRP levels were detected before fever under weekly CRP monitoring, dose adjustment of clozapine during the detection of elevated CRP levels may have prevented the onset of fever. Furthermore, of the 10 patients with no fever during weekly CRP monitoring, three (Cases 8, 9, and 10) had CRP level elevations >3.0 mg/dL and halting an increase in the clozapine dose from that point may have prevented the onset of fever. Therefore, weekly CRP monitoring detected elevated CRP levels in 5 of the 21 patients under asymptomatic conditions. CRP determination could be clinically worthwhile in more asymptomatic inflammatory conditions, considering the myocarditis that occurred in 20% without fever (10). Including cases of fever, CRP increased during clozapine administration in almost all cases and decreased when clozapine was stopped or reduced. The findings, in general, point to the inflammatory properties of clozapine (for the majority of people) and make CRP, in itself, a non-specific marker, sensitive, but non-specific for detecting dangerous inflammation. Clinicians should adjust clozapine doses during clozapine initiation and pay attention to physical findings, vital signs, and laboratory findings suggestive of fever and inflammation.

### 4.2 The clinical utility of daily CRP monitoring during clozapine titration

Daily CRP monitoring during clozapine titration has been adopted in Australia when the CRP level is >5 mg/dL or the troponin level is above the upper normal limit to prevent clozapine-induced myocarditis (9, 10). Clozapine discontinuation is recommended when the CRP level is >10 mg/dL or the troponin level is greater than twice the upper limit of normal. As our two cases of fever with daily CRP measurements showed, daily measurement of CRP from the onset of clozapine-induced inflammation can provide information on the severity of inflammation and can be used as a guide for the early adjustment or resumption of clozapine doses. Measuring CRP daily during the period before the onset of clozapine-induced inflammation is more sensitive to detecting elevated CRP levels than measuring CRP weekly. However, considering the patient burden and cost, weekly CRP measurement in conjunction with the mandated weekly neutrophil count may be a more equitable approach. Implementing a device that utilizes finger capillary blood sampling (as has been performed in this study) to measure CRP levels, specifically during high-risk periods such as day 10 through day 20, would be an efficient way of alleviating the burden on patients. Intensive monitoring of several parameters is perceived as safe but may be burdensome to patients and caregivers in the use of clozapine; therefore, monitoring should be balanced according to ethnicity, concomitant medications, titration speed, and other risks. Nevertheless, as this study showed, weekly CRP monitoring may not predict clozapine-induced inflammation in some cases, and clinicians should be alert to the sudden onset of clozapine-induced inflammation, even when the CRP test is negative.

In future studies, the analysis of daily CRP trends during clozapine titration in several cases may reveal patterns that predict clozapine-induced inflammation. In our two cases with fever and daily CRP measurements, a pattern of mildly elevated CRP levels was observed a few days before fever onset.

### 4.3 Limitations

This study was a case series of 21 Japanese patients that aimed to present patterns of CRP trends during clozapine titration. A limitation of this study was the small number of participating patients, especially those who were assessed on a daily basis (only 4). Therefore, the benefits of determining CRP daily versus once a week need to be demonstrated by independent studies with more prominent subjects. Prospective studies are needed to investigate how clozapine doses can be adjusted to prevent the development of clozapine-induced inflammation when CRP levels reach a certain threshold.

In conclusion, weekly and daily CRP monitoring during clozapine titration is valuable in preventing clozapine-induced inflammation, assessing its severity, and guiding clozapine dose adjustments. However, weekly CRP monitoring may not adequately predict clozapine-induced inflammation in some cases, and clinicians should be aware of the sudden onset of clozapine-induced inflammation, even if CRP levels are unremarkable. Daily CRP monitoring is better for detecting clozapine-induced inflammation.

## Data availability statement

The original contributions presented in the study are included in the article/**Supplementary Material**. Further inquiries can be directed to the corresponding author.

## Ethics statement

The studies involving humans were approved by Tohoku University Hospital Ethics Review Board. The studies were conducted in accordance with the local legislation and institutional requirements. Written informed consent for participation in this study was provided by the participants’ legal guardians/next of kin. Written informed consent was obtained from the individual(s), and minor(s)’ legal guardian/next of kin, for the publication of any potentially identifiable images or data included in this article.

## Author contributions

YK: Conceptualization, Formal Analysis, Investigation, Methodology, Project administration, Writing – original draft, Writing – review & editing. HTa: Conceptualization, Investigation, Methodology, Writing – review & editing. SU: Investigation, Writing – review & editing. YO: Investigation, Writing – review & editing. MG: Investigation, Writing – review & editing. MI: Investigation, Writing – review & editing. TT: Investigation, Writing – review & editing. HK: Supervision, Writing – review & editing. HTo: Supervision, Writing – review & editing.
