# Supplementary material for: Patterns of C-reactive protein trends during clozapine titration and the onset of clozapine-induced inflammation: a case series of weekly and daily C-reactive protein monitoring
Source: Front Psychiatry. 2024 Feb 21;15:1366621. doi: 10.3389/fpsyt.2024.1366621 (PMC10914995; doi:10.3389/fpsyt.2024.1366621)
Supplement: Supplementary file 1 [file Table_1.docx]

Supplementary Material

Patterns of C-reactive protein trends during clozapine titration and the onset of clozapine-induced inflammation: A case series of weekly and daily C-reactive protein monitoring

Yuki Kikuchi ^1, 2, *^, Hiroaki Tanifuji ^3^, Sota Ueno ^2^, Yoshifumi Onuma ^2^, Masatomo Goto ^2^, Masato Ishihara ^2^, Takeshi Toraiwa ^2^, Hiroshi Komatsu ^4^, Hiroaki Tomita ^1, 4^

^1^ Department of Psychiatry, Graduate School of Medicine, Tohoku University, Sendai, Miyagi, Japan

^2^ Department of Psychiatry, Kodama Hospital, Ishinomaki, Miyagi, Japan

^3^ Department of Pharmacy, Kodama Hospital, Ishinomaki, Miyagi, Japan

^4^ Department of Psychiatry, Tohoku University Hospital, Sendai, Miyagi, Japan

*** Correspondence:**Yuki Kikuchi.

Email: ykikuchi@sand.ocn.ne.jp

# Supplementary Tables

| **Supplementary Table 1.** The clinical characteristics and psychopathological descriptions of the patients | | | | | |  |  |
| --- | --- | --- | --- | --- | --- | --- | --- |
| Case | Age | Sex | Diagnosis (DSM-5) * | Duration of Illness until clozapine initiation (year) | Severe psychopathological dimensions at the start of clozapine administration | Continuation of clozapine as of January 2024 |  |
| 1 | 48 | F | Schizophrenia | 28 | Positive symptoms Disorganization | Yes |  |
| 2 | 62 | F | Schizophrenia | 32 | Positive symptoms Disorganization Cognitive deficits | Yes |  |
| 3 | 43 | M | Schizophrenia | 24 | Positive symptoms Disorganization | Yes |  |
| 4 | 36 | M | Schizophrenia | 19 | Positive symptoms Negative symptoms | Yes |  |
| 5 | 22 | M | Schizophrenia | 5 | Positive symptoms Mood symptoms (Depression, anxiety, suicidal thoughts) | Yes |  |
| 6 | 50 | M | Schizoaffective disorder | 20 | Mood symptoms (Depression, anxiety, suicidal thoughts) | Yes |  |
| 7 | 42 | M | Schizophrenia | 19 | Positive symptoms Disorganization | Yes |  |
| 8 | 52 | M | Schizophrenia | 24 | Positive symptoms Mood symptoms (Depression, anxiety, suicidal thoughts) | Yes |  |
| 9 | 33 | F | Schizophrenia | 17 | Positive symptoms Disorganization | Yes |  |
| 10 | 24 | M | Schizophrenia | 1 | Positive symptoms Disorganization | Yes |  |
| 11 | 69 | F | Schizophrenia | 48 | Positive symptoms Cognitive deficits | Yes |  |
| 12 | 60 | M | Schizophrenia | 40 | Positive symptoms Disorganization | Yes |  |
| 13 | 25 | M | Schizophrenia,  Autism spectrum disorder | 1 | Positive symptoms Disorganization | Yes |  |
| 14 | 43 | F | Schizophrenia | 9 | Positive symptoms | Yes |  |
| 15 | 35 | F | Schizophrenia | 2 | Positive symptoms Disorganization | Yes |  |
| 16 | 37 | F | Schizophrenia | 19 | Positive symptoms Disorganization | Yes |  |
| 17 | 30 | M | Schizophrenia,  Autism spectrum disorder | 15 | Positive symptoms Disorganization | Yes |  |
| 18 | 49 | M | Schizophrenia | 28 | Positive symptoms | Yes |  |
| 19 | 17 | F | Schizophrenia | 1 | Positive symptoms Mood symptoms (Depression, anxiety, suicidal thoughts) | Yes |  |
| 20 | 42 | F | Schizophrenia | 6 | Positive symptoms | Yes |  |
| 21 | 46 | M | Schizophrenia | 31 | Positive symptoms Disorganization | Yes |  |
| * All patients were diagnosed with treatment-resistant schizophrenia, treated with two or more second-generation antipsychotics (chlorpromazine equivalent ≥ 600 mg/day) for ≥ 4 weeks and had never scored > 41 on the global assessment of functioning. | | | | | | |  |
|  |  |  |  |  |  |  |  |

| **Supplementary Table 2.** CRP trends after day 30 in two cases with daily CRP measurements | | | | | | | | | | | |  |  |  |  |  |  |  |  |  |  |  |  |  |  |  |  |
| --- | --- | --- | --- | --- | --- | --- | --- | --- | --- | --- | --- | --- | --- | --- | --- | --- | --- | --- | --- | --- | --- | --- | --- | --- | --- | --- | --- |
| Case |  | Day | 30 | 31 | 32 | 33 | 34 | 35 | 36 | 37 | 38 | 39 | 40 | 41 | 42 | 43 | 44 | 45 | 46 | 47 | 48 | 49 | 54 | 60 | 61 | 68 | |
| 18 |  | CRP | 0.2 | 0.2 | 0.3 | 0.2 | 0.2 | 0.1 | 0.1 | 0.1 | 0.2 | 0.1 | 0.1 |  |  |  |  |  |  | 0.5 |  |  | 0.2 |  | 0.1 | 0.3 | |
|  |  | BT | 36.6 | 36.7 | 36.6 | 36.1 | 36.4 | 36.1 | 36.3 | 36.3 | 36.5 | 36.4 | 36.3 |  |  |  |  |  |  | 36.3 |  |  | 36.6 |  |  | 36.6 | |
|  |  | Dose | 50 | 50 | 50 | 50 | 50 | 50 | 75 | 75 | 75 | 75 | 75 |  |  |  |  |  |  | 100 |  |  | 125 |  | 150 | 175 | |
|  |  |  |  |  |  |  |  |  |  |  |  |  |  |  |  |  |  |  |  |  |  |  |  |  |  |  | |
| 19 |  | CRP | 0.7 | 0.4 | 0.5 | 0.5 | **1.2** | **1.7** | **2** | 0.9 | **1.7** | **3.2** | **5.3** | **2.7** | **1.3** | 0.9 | 0.5 | 0.4 | 0.3 | 0.2 | 0.2 | 0.2 | 0.4 | **1.0** | 0.2 |  | |
|  |  | BT | 36.7 | 37.0 | 37.0 | 37.0 | 37.1 | 37.1 | 37.2 | 36.9 | 36.4 | 37.4 | 36.8 | **37.5** | 36.8 | 36.7 | 36.8 | 36.9 | 36.5 | 36.4 | 36.9 | 37.0 |  |  |  |  | |
|  |  | Dose | 75 | 75 | 75 | 100 | 100 | 100 | 100 | 100 | 100 | 100 | 50 | 50 | 50 | 50 | 75 | 75 | 75 | 75 | 75 | 75 | 75 | 75 | 75 |  | |
| Units of measurement: CRP (mg/dL), BT(°C), and Dose (mg). CRP levels of 1.0 mg/dL or higher and BT of 37.5°C or higher are shown in bold. | | | | | | | | | | | | | | | | | | | | | | | | | | |  |
| Abbreviations: BT, body temperature; CRP, C-reactive protein. | | | | | | |  |  |  |  |  |  |  |  |  |  |  |  |  |  |  |  |  |  |  |  |  |
